# Natural C-independent expression of restriction endonuclease in a C protein-associated restriction-modification system

**DOI:** 10.1093/nar/gkv1331

**Published:** 2015-12-09

**Authors:** Monika Rezulak, Izabela Borsuk, Iwona Mruk

**Affiliations:** Department of Microbiology, University of Gdansk, Wita Stwosza 59, 80-308 Gdansk, Poland

## Abstract

Restriction–modification (R-M) systems are highly prevalent among bacteria and archaea, and appear to play crucial roles in modulating horizontal gene transfer and protection against phage. There is much to learn about these diverse enzymes systems, especially their regulation. Type II R-M systems specify two independent enzymes: a restriction endonuclease (REase) and protective DNA methyltransferase (MTase). Their activities need to be finely balanced *in vivo*. Some R-M systems rely on specialized transcription factors called C (controller) proteins. These proteins play a vital role in the temporal regulation of R-M gene expression, and function to indirectly modulate the horizontal transfer of their genes across the species. We report novel regulation of a C-responsive R-M system that involves a C protein of a poorly-studied structural class - C.Csp231I. Here, the C and REase genes share a bicistronic transcript, and some of the transcriptional auto-control features seen in other C-regulated R-M systems are conserved. However, separate tandem promoters drive most transcription of the REase gene, a distinctive property not seen in other tested C-linked R-M systems. Further, C protein only partially controls REase expression, yet plays a role in system stability and propagation. Consequently, high REase activity was observed after deletion of the entire C gene, and cells bearing the ΔC R-M system were outcompeted in mixed culture assays by those with the WT R-M system. Overall, our data reveal unexpected regulatory variation among R-M systems.

## INTRODUCTION

Many species of bacteria and archaea possess restriction-modification (R-M) systems (1,2) that, among other roles, serve to resist bacteriophage predation and to modulate gene flow (3,4). R-M systems are classified into four main types, with Type II numerically dominating Types I, III and IV (5). A type II R-M system is typically composed of two independent enzymes: a restriction endonuclease (REase) that cleaves DNA at a specific sequence, and a modification methyltransferase (MTase) that acts on the same sequence to protect it from cleavage by the cognate REase. This gives a simple mechanism for discriminating between self- and non-self DNA (6,7) in that the REase can degrade DNA entering the cell, while resident host DNA remains uncleaved due to methylation by the MTase. Type II R-M system activity may be harmful to its host, when expression of the REase and MTase is not finely balanced. Toxic REase action may cause double-strand breaks in a genome, and it can be lethal for the host if unrepaired (8,9). It resembles the functioning of many toxin-antitoxin units and other genetic addiction elements, which demand a counterbalance to toxicity to avoid the post-segregational killing of the host (10–12). Since R-M systems are highly abundant and mobile within Prokaryotes, (5,13) some mechanisms must exist to provide the coordinated, temporal control of REase expression. It is especially crucial during the transfer of an R-M system to a new host cell wherein, the genome is unprotected by methylation. A sufficient strategy employed is to delay REase expression to allow the MTase first to complete genome methylation (14). The molecular basis of these processes is still enigmatic, and it is understood only to some extent for Type II R-M systems. It seems likely that they require fine-tuned transcriptional feedback circuits to keep the REase/MTase activities in balance.

Three control mechanisms for Type II R-M system expression have been outlined: MTase, antisense RNAs, and C proteins, but none of these mechanisms is understood in great detail. In the first case, a MTase represses its own transcription via cognate operator binding within the promoter (15–17). Additionally, in some R-M systems the MTase recognition sequence for modification is located within its own gene's promoter, and the methylated promoter sequence represses MTase gene expression. Eventually the low level of enzyme leads to loss of methylation after replication and promoter activity is unblocked (18,19). Such feedback loops operate to prevent MTase overexpression, but do not explain the control of toxic REase expression. For the Ecl18kI R-M system, the control of expression is exerted at the level of transcription kinetics when MTase autorepression is accompanied by an additional promoter competition mechanism to ensure the MTase/REase expression is balanced (20).

The second mechanism of coordinated expression of R-M system seems to apply to bicistronic systems where the REase gene precedes the MTase, as in EcoRI and Eco29kI (21–24). In these cases antisense RNAs produced from oppositely-oriented promoters negatively regulate REase and MTase expression (23,24). Weakening or knocking out an antisense promoter results in enhancement of REase expression to the point of toxic enzyme accumulation and cell death (23). In contrast, overexpression of antisense RNA *in trans* alleviates REase toxicity after loss of the R-M system (23).

The third important mode of R-M system expression control relies on a specialized protein transcription factors called C proteins. These controller proteins are present in more than 300 R-M systems, and were first discovered in the PvuII and BamHI R-M systems (25,26). Typically, a C gene precedes an REase gene and sometimes partially overlaps it (26–35). The upstream location of C facilitates efficient and precise C-dependent transcriptional control over the toxic REase gene (35–38). In one tested exception to this paradigm, Kpn2I, the C gene precedes and controls the MTase gene, but has no effect on REase gene expression (39). In one of the best-studied C-dependent R-M systems, PvuII, the C and REase genes share the same bicistronic transcript controlled by both a weak C-independent promoter and a stronger C-dependent promoter (29,36,40). If C protein is inactivated or absent then REase expression is very low, and REase activity is undetectable. If the C gene is supplied *in trans* then REase levels return to the wild-type values (29,34). The C/REase expression level is a result of a gene-copy-dependent feedback loop that activates transcription at low C protein concentrations and represses when levels become high (36,37,41). To exert control C protein binds to a specific palindromic DNA sequence (C-box) that is embedded in its own promoter region. In the Esp1396I R-M system, there is a C-box upstream of the C gene, and another upstream of the MTase gene; each has distinct C binding affinities (38). In another case, the C protein is translationally fused to the REase, and it controls the expression of the fused gene (42). More generally, C proteins are grouped into three informal prototype families (C.PvuII, C.EcoRV and C.EcoO109I) based on the conservation of the C-box nucleotide sequence. The C.EcoO109I family has been studied the least (36), and more systematic detection of inverted repeats/palindromes upstream of C genes has identified several additional motifs (43,44).

This study focuses on a new regulatory class of C protein-associated R-M systems with the C protein (C.Csp231I) being a member of the understudied C.EcoO109I family. A crystal structure for C.Csp231I is available (45,46), but *in vivo* regulatory studies have not been performed for related R-M systems except in the C-box class prototype EcoO109I (33). We find that the C protein role in Csp231I is distinct from its role in EcoO109I, and we report a novel regulatory mode for a C protein-associated R-M system with separate promoters for the C and REase genes. We further demonstrate high REase activity, regardless of C gene presence, which is unlike the case with other characterized C proteins. We do discuss the possible role that C.Csp231I plays in regulating its cognate R-M system, but in general we find that much remains to be learned even about this subset of C-controlled R-M systems.

## MATERIALS AND METHODS

### Strains, plasmids and oligonucleotides

The source of studied R-M system was *Citrobacter* sp. RFL231, and was kindly supplied by Dr. A. Janulaitis, MBI Fermentas, Lithuania. Despite the fact the *E. coli* and *Citrobacter* are both members of the *Enterobacteriaceae*, expression of the Csp231I R-M system in *E. coli* cells seemed to be toxic. Accordingly, to clone the WT Csp231I R-M system the competent cells were prepared from *E. coli* MM294 strain, which expresses the MTase gene from pEcoVIIIM to ensure the protection of host genome. M.Csp231I and M.EcoVIII both recognize the same nucleotide sequence (47,48). Other *E. coli* K-12 strains used in this study are described below. MC1061 [*araD139* Δ(*ara, leu*)7697, Δ*lacX74, galU, galK, hsdR, strA*] was used in all *lacZ* reporter assays (49). *E. coli* DH5α and MM294 were used for all other purposes including cloning steps. *E. coli* Rosetta was used for C protein overproduction and purification (50). The plasmids used are listed in Supplementary Table S1. They were also deposited in the Collection of Plasmids and Microorganisms, University of Gdansk, Poland. The oligonucleotides used are shown in Supplementary Table S2 of the Supplementary Data.

### Effect of C gene delivered *in trans*

The gene for C.Csp231I or its variant was PCR-amplified and cloned downstream of the arabinose-inducible P_araBAD_ promoter in vector pBAD24 or pBAD33 (51) yielding series of wildtype (pBAD-CWT) and variant (pBAD-arq and pBAD-sqe) plasmids (Supplementary Table S1). Arabinose induction experiments were performed in M9-minimal medium with 0.2% glycerol as the carbon source (52). Briefly, single colonies were used to inoculate overnight cultures in M9 media supplemented with appropriate antibiotics. These cultures were diluted 1:50 into antibiotic free medium, and grown with shaking to an OD_600_ of 0.2–0.3. The cells were then gently pelleted, resuspended, and divided among flasks containing M9-minimal media with varied concentrations of L-arabinose. After about 3 hours of subsequent cultivation, ONPG assay was performed and Miller or modified Miller units were calculated as previously described (23,36,53). A series of transcriptional *lacZ* fusions were generated in the pRS415 vector (54), and translational *lacZ* in-frame fusions were created using pLex3B (55). Details regarding plasmid cloning and features are outlined in the Supplementary Data (Supplementary Table S1).

### RNA isolation, RT-PCR and determination of transcription start points

*E. coli* carrying p18 plasmid with WT Csp231I R-M system were grown to exponential phase and pelleted. Total cellular RNA was isolated using the Total RNA Kit (A&A Biotechnology, Poland). The transcription start points of the genes encoding the Csp231I R-M system were determined by the primer extension method. The 5′ ends of appropriate primers were labeled with 5 pmol of [*γ*-^32^P]ATP (primer EXMET for mapping P_M_; primer EXRES for mapping P_R_ and C16 for mapping P_C_). Twenty-μl primer extension reactions containing 10 μg RNA, 0.6 pmol of labeled primer, buffer (50 mM Tris-HCl pH 8.3, 50 mM KCl, 4 mM MgCl_2_, 10 mM DDT), 1 mM of each dNTPs and 10 u RiboLock RNase Inhibitor were denatured at 95°C for 3 minutes, and then incubated at 50°C for 1 hour. Next, 200u RevertAid H Minus Reverse Transcriptase (Fermentas) was added and samples were incubated at 42°C for 30 min. Finally, 4 μl of loading dye (95% formamide, 0.05% bromophenol blue, 0.05% xylene cyanol) was added, samples were denatured at 75°C and then loaded onto 6% acrylamide:bis (19:1)–7 M urea in 1× TBE gel (52). Sequencing reactions were also performed on DNA templates using the DNA Cycle Sequencing Kit (Jena Bioscience), and the appropriate radiolabelled primer. These samples were loaded on the acrylamide gels with the primer extension reactions.

For reverse transcription PCR (RT-PCR), 5 μg of total RNA was DNase I treated in solution at 37°C for 1 h using the RNAse-free DNase I (Eurx, Poland)). After a 20 min inactivation at 65°C, the cDNA synthesis was performed using RevertAid H Minus Reverse Transcriptase (Fermentas) kit with random hexamers according to the manufacturer's protocol. The resultant cDNA was then used as template for PCR with indicated primer pairs. The resultant PCR products were separated on 2% TBE agarose gels.

### Electrophoretic mobility shift assays (EMSA)

DNA specific substrate was double-stranded PCR-amplified (primers: C20–C24; p18 plasmid as a template, Supplementary Tables S1 and S2) fragments that were 576 bp in length and included the entire P_C_ promoter/operator region. The non-specific DNA substrate (515 bp) containing the P_M_ promoter region was amplified with C2-C30 primers. Reactions containing 100 nM DNA and the indicated protein concentrations were prepared in binding buffer [50 mM Tris–HCl (pH 8.0), 1 mM DTT, 10 mM MgCl_2_] to a final volume of 20 μl, and incubated for 20 min at 22°C. Samples were electrophoresed on 6% native polyacrylamide gels in 0.5× TBE buffer at 22°C. The location of dsDNA in the gels was determined by ethidium bromide staining.

### Overproduction and purification of the C protein and its variants

The coding sequence of C gene was PCR-amplified from *Citrobacter* sp. RFL231 genomic DNA using primers CNco and CRev. NcoI and EcoRI treated PCR fragments were cloned into pET28(+) linearized with the same restriction enzymes to generate pET-CWT, which produces a C protein with a C-terminal His5-tag. The other C gene variants were generated using Quick-Change mutagenesis on the pET-CWT template. Resultant plasmids were: pET-Csqe (primers sqe1 and sqe2) producing a C protein variant with the following triple amino acid residue substitution S16A; Q17A; E18A, and pET-Carq (primers arq1 and arq2) producing the A33G; R34E; Q37A C protein variant. The vectors were sequenced confirmed and are called C-SQEmut and C-ARQmut respectively throughout the text.

For protein purification, the host *E. coli* Rosetta strain was used to overexpress all three His5-tagged C protein variants (50). Cells were grown in 100 ml LB broth supplemented with appropriate antibiotics at 37°C. At an OD_600_ of 0.3 C protein production was induced by adding IPTG to 0.5 mM. After a 3 hour incubation, cells were pelleted and stored at −70°C until used. Frozen cells were thawed in C buffer (50 mM NaH_2_PO_4_, 300 mM NaCl, 10 mM imidazole, 5% glycerol, 1 mM PMSF and 5 mM β-ME pH 8.0) and sonicated (40 × 10 s). The lysates were cleared by centrifugation and applied to a column packed with TALON cobalt resin (Clontech), and washed with buffer C. The bound proteins were eluted with 150 mM imidazole in C buffer. Protein-containing fractions were pooled, and dialysis was performed over night in buffer containing 50 mM MgCl_2_, 5% glycerol, 50 mM Tris–HCl pH 8.0 to remove excess of imidazole. Finally, purified protein samples were concentrated in 50% glycerol and stored at –20°C. The protein concentration was determined by densitometry in Tricine–SDS-PAGE (56) using a lysozyme as a quantitative standard.

### Western blot analysis

Samples of cultures containing similar number of cells were centrifuged, supernatants were removed, and the cell pellets stored at –80°C. Pellets were resuspended in 1× SDS Laemmli buffer (52), and lysed by heating at 98°C for 10 min. Proteins were resolved by Tricine-SDS PAGE (56) and then electroblotted to PVDF membranes. C.Csp231I protein bands were detected by chemiluminescence using the ECL-plus Western Blotting Detection System (GE Health Sciences) with 1:2000 dilution of rabbit anti-C.Csp231I polyclonal serum prepared according to standard protocols (57), and a 1:30 000 dilution of horseradish peroxidase-conjugated goat anti-rabbit IgG. Protein bands were visualized either by autoradiography or by using 5-bromo-4-chloro-3-indolylphosphate (BCIP) as the alkaline phosphatase substrate and nitroblue tetrazolium (NBT) as the color development reagent. The prestained MW markers used were PageRuler (Fermentas).

### Efficiency of transformation assay

Efficiency of transformation (EOT) is defined in this study as the relative number of transformants obtained from a given preparation of competent cells using a non-saturating amount of plasmid DNA. EOT is calculated from the ratio of transformants with a test plasmid relative to those with the control vector. This term is equivalent to the term ‘relative transformation efficiency’. In this particular case due to toxicity of the investigated R-M system, *E. coli* cells MM294 carrying plasmid with the protective MTase gene [pEcoVIIIM] were used as a competent cells. The standard CaCl_2_-heat shock method of transformation was used (52).

### Relative restriction activity assay

The restriction activity of *E. coli* cells carrying Csp231I R-M system and its variants was measured by determining plating efficiency of bacteriophage λ*vir*. The efficiency of plaquing (plaque-formation) (EOP) of λ*vir* was calculated as the ratio of plaques formed on *E. coli* MM294 [pEcoVIIIM] containing plasmid pBR322 (restriction negative) to those formed on the same strain containing a plasmid with the Csp231I R-M system or its variants. Relative restriction ( = 1) refers to the WT R-M system.

### Fitness assay by mixed culture competition experiment

Five ml of M9-glucose medium without any antibiotic was inoculated with two comparably sized colonies of *E. coli* MG1655 obtained from a fresh transformation grown on LB-agar plates with appropriate selective antibiotic. One colony carried the p18amp plasmid bearing the WT R-M system, and was selective on ampicillin. The second colony, selected on tetracycline, contained its variant - the p30tet plasmid wherein the C gene and its C-box sequence were deleted. The *bla* gene of p30tet was disrupted to change the plasmid selection marker to tetracycline resistance. In parallel control cultures, plasmids with restriction-negative and modification-positive variants (p17amp versus p31tet) or empty vectors (pBRamp versus pBRtet) were used. Time zero of the competition experiment marks the point at which 1:1 mixed cultures of competing cells were inoculated. Every 15–18 hrs of incubation at 37°C with shaking, the co-cultures were diluted 10^6^ into fresh minimal media without antibiotics. A sample of each mixed competition culture was immediately taken, appropriately diluted and spread quantitatively either onto LB-agar containing the appropriate selective antibiotic or on antibiotic free LB agar. The colonies were counted and the ratio of colony-forming units (CFU) of the two competing cell populations was calculated using *T* = (CFU_amp_/CFU_tet_); their generation number was also determined. Data were normalized using the results from the vector control (*T* = (CFU_amp_/CFU_tet_)). The relative competitive fitness (*W*) was then calculated as *W* = log[*T*/*V*] for each tested generation time-point (58).

## RESULTS

As reported here, the Csp231I R-M system was cloned from chromosomal DNA of *Citrobacter* sp. RFL231, by selecting for the MTase. We used a suicide plasmid that carried a functional REase gene (no MTase gene), from which cells would be protected if they had a MTase with corresponding specificity (59). The recognition sequence (5′-AAGCTT-3′) is the same as the prototype HindIII R-M system (60) as well as several other isospecific systems studied in our laboratory: EcoVIII from *E. coli* E1585–68 (47,48,61); LlaCI from *Lactococcus lactis* subsp. *cremoris* W15 (62,63) and BstZ1II from *Bacillus stearothermophilus* 14P (59).

**Figure 1.**
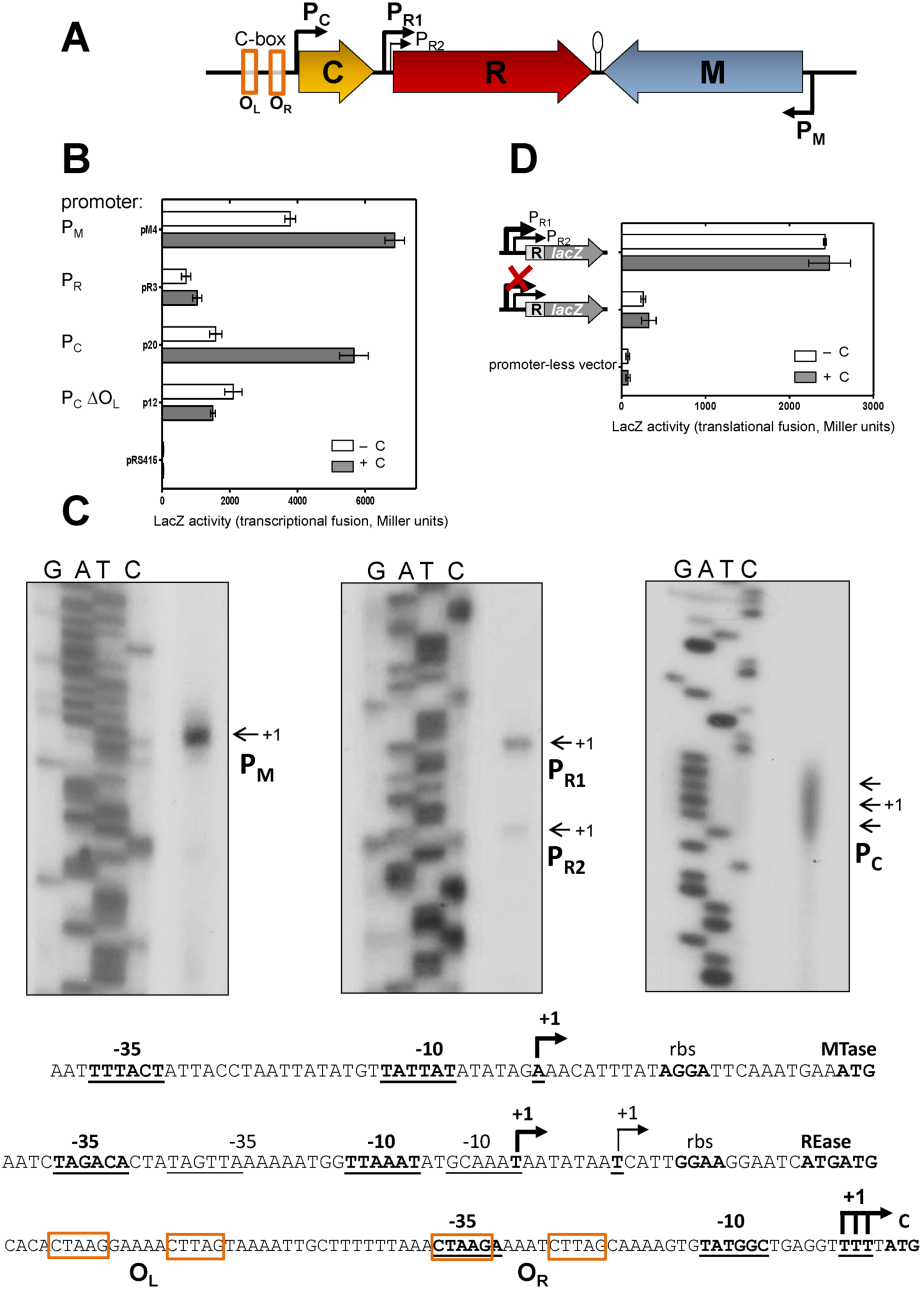
Csp231I R-M system and its transcription initiations. (**A**) Genetic organization of the Csp231I R-M system comprising of its regulator (C gene and its promoter P_C_), REase (R gene and its two promoters: major P_R1_ and minor P_R2_) and MTase (M gene and its P_M_) (not to scale). The identified promoters are designated by arrows. The operator for P_C_ promoter, C-box, consists of two inverted repeats CTAAG-n_5_-CTTAG, left and right, marked as O_L_ and O_R_ respectively. A presence of Rho-independent transcription terminator in the 152-nt intergenic region separating R and M genes is predicted and depicted here as hairpin. (**B**) Identified promoters activity was measured as a transcriptional fusion of appropriate DNA fragments with indicated promoter to the reporter *lacZ* gene. In case of P_C_, deletion of left operator (ΔO_L_) also has been tested. The transcription activity in context of C protein absence (vector control, white bars) or C protein presence (pBAD-CWT, dark bars) is presented in Miller units. The results are the averages (±SD) of at least three independent experiments. (**C**) Mapping the transcription start sites for the promoters with confirmed activity. For each reaction, total RNA from *E. coli* harboring the p18 plasmid with entire, functional R-M system was used as template for primer extension method using radioactively labeled primers and performed as indicated in Materials and Methods. The primer extension products (marked as +1) were resolved on a denaturing 6% polyacrylamide gel along with the nucleotide sequencing reactions (G, A, T, C) performed with the same labeled primer and appropriate DNA template. At the bottom, sequence of relevant DNA containing the indicated promoters is also shown. The -10 and -35 promoter motifs are underlined and the start codons (ATG) and ribosome binding sites are in bold. In case of P_C_ promoter, the sequence of two inverted repeats CTAAG-n_5_-CTTAG is boxed. For REase promoters, the start of transcription for major promoter P_R1_ is indicated by thicker arrow and bold -10 and -35 boxes, in contrast to the minor P_R2_ promoter marked by thin arrow. (**D**) Promoter activity for REase gene was tested by ONPG hydrolysis in plasmid constructs of pLEX3B, where reporter *lacZ* gene was fused in-frame to REase gene (pLEX-P_R1_WT). The major P_R1_ promoter (thicker bent arrow) was knocked-out by mutation of -10 box of TTAAAT→CCCGGG (pLEX-P_R1_mut). The transcription activity in context of C gene presence or absence was measured as in panel B.

The genetic organization of Csp231I R-M system is shown in Figure 1A. In addition to the convergently oriented REase (*csp231IR*) and MTase (*csp231IM*) genes, the regulatory C gene (*csp231IC*) is present. The *csp231IC* gene is upstream of *csp231IR* in a typical colinear location. The genes do not overlap, unlike other C and REase genes (31,64), which reduces the possibility of translational coupling. Moreover, the sequence analysis indicated a potential Rho-independent transcription terminator in the 152-nt intergenic region separating *csp231IR* and *csp231IM* genes (www.softberry.com).

### Each gene of the Csp231I R-M system has its own promoter

To determine the localization of promoters, we cloned the upstream regions of the three Csp231I R-M system genes into the pRS415 vector upstream of a promoterless *lacZ* reporter gene. LacZ enzyme activity was measured via ONPG hydrolysis assays. Promoter activity was detected for all three DNA fragments in: pM4 (P_M_ of MTase gene), p20 (P_C_ of C gene) and pR3 (P_R_ of REase gene) (Figure 1B; white bars). The latter result was unexpected as REase genes in C protein-associated R-M systems typically rely on a shared promoter upstream of C gene (29,36,40).

Next, we tested whether the detected promoter activities change when C.Csp231I is delivered *in trans* (Figure 1B; dark gray bars). The C gene was cloned into a compatible plasmid under a P_araBAD_ promoter to generate pBAD-CWT. The resulting LacZ activities revealed a positive regulatory effect of C protein on both the P_M_ and P_C_ promoters, but surprisingly not on P_R_ (Figure 1B; black bars). The P_C_ and P_M_ promoter activity increased about 3.5 and 2.0-fold, respectively. The regulatory effect of a C protein is related to its binding to inverted repeats (C-box; motif 8; (43)) located usually within the promoter region. We found two such repeats and deleted the left part (O_L_) generating plasmid p12. The promoter activity this variant remained unchanged regardless of the C protein presence or absence. These results indicate there is at least one promoter that can be stimulated for higher activity (directly or indirectly) by C protein. In the putative promoter for the MTase gene (P_M_), no inverted repeats have been detected. A similar observation of the C protein effect on MTase expression of unclear origin was reported previously (33).

To localize the promoters, we identified transcription start sites of Csp231I genes by primer extension, using total RNA prepared from *E. coli* cells carrying the complete, functional Csp231I R-M system. For the MTase gene, a single primer extension product was produced (Figure 1C). Probable appropriately positioned –10 (TATTAT) and –35 (TTTACT) sequences were identified based on comparison to *E. coli* consensus sequences (TATAAT and TTGACA with 17nt spacer, respectively (65)). For the REase gene, two primer extension products were found with one being much more intense, and so assumed to be the major promoter (P_R1_; Figure 1C). The identified -10 (TTAAAT) and -35 elements (TAGACA) of P_R1_ each showed only a single nucleotide difference from the consensus sequences. The minor P_R2_ promoter revealed –10 and –35 elements as GCAAAT and TAGTTA. To test the REase promoter identification the TTAAAT -10 box of the major P_R1_ promoter was changed to CCCGGG (pLEX-P_R1_WT versus pLEX-P_R1_mut) (Figure 1D), and this drastically decreased transcription activity regardless of C gene presence *in trans*. However, the remaining activity was still slightly above the promoterless level (pLex3B) probably indicating weak activity from the second P_R2_ promoter (Figure 1D).

Mapping the start of mRNA for the C gene showed a single spot of multiple products within a stretch of five ‘T's located on the DNA template just upstream of the translational start (Figure 1C). This indicates that the transcript is leaderless (the start codon is either preceded by only a few nucleotides, as in this case, or it starts directly with a 5-terminal AUG) much like many homologous regulatory C genes (27,35,40). The multiple products from the primer extension most likely result from reiterative transcription that may occur during transcription initiation, and can impact gene expression in some cases (66,67). Nevertheless, we found these sequences suboptimal to the consensus sequence: CTAAGA (–35 box) and TATGGC (–10 box). Since we considered a second transcript for the C gene to be possible, we carefully mapped a start site on RNA isolated from cells carrying the p20 reporter gene in the presence and absence of C (as in Figure 1B), and each time the same products within a single cluster were obtained (Figure 1C and data not shown). We also confirmed the absence of other transcripts for the C gene by reverse transcription PCR and it indicated that no mRNA is produced from the sequence upstream of –10 position of P_C_ promoter (Supplementary Figure S1).

### C protein specifically binds its C-box sequence *in vitro*

**Figure 2.**
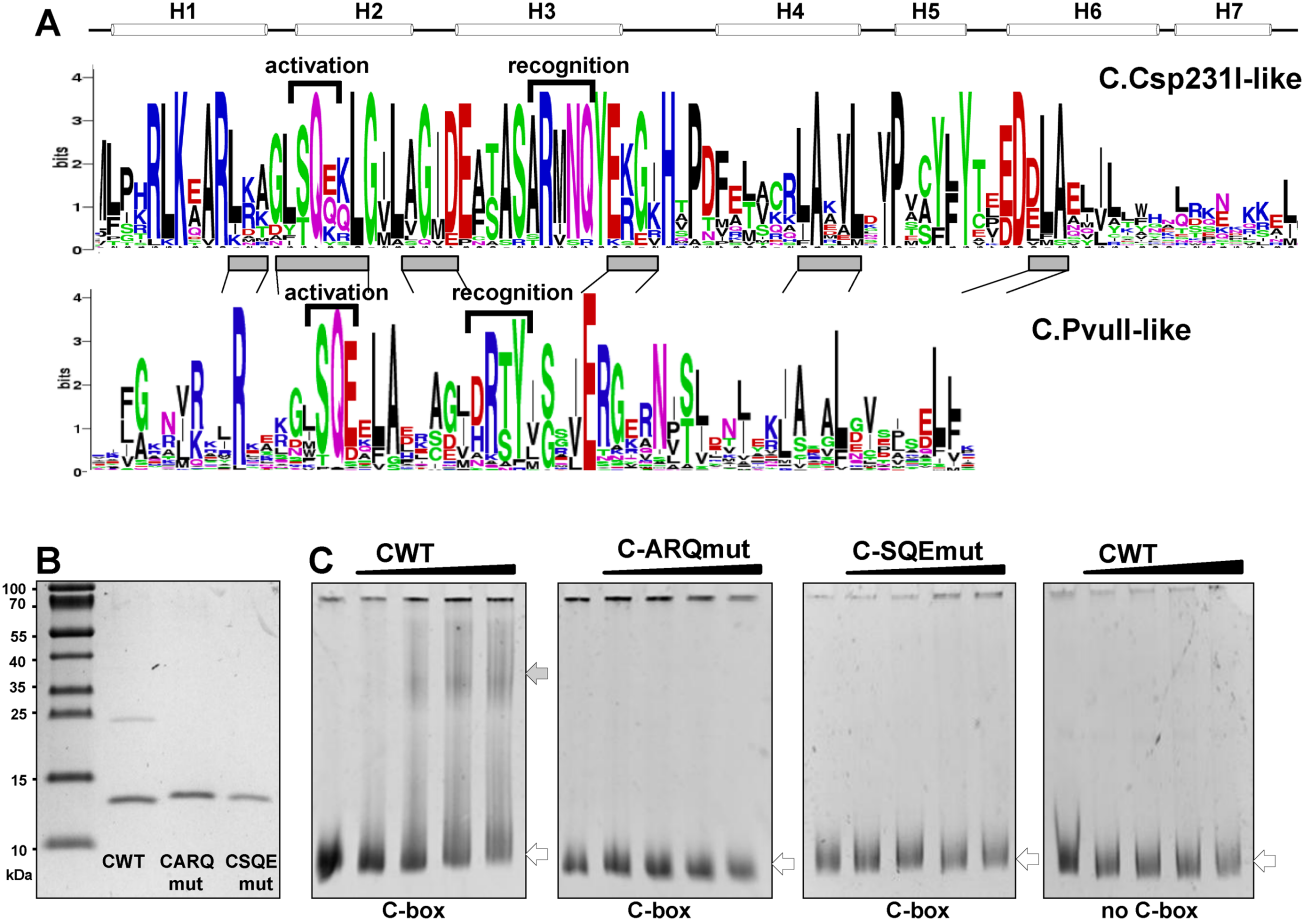
C.Csp231I protein and its action *in vitro*. (**A**) LOGO of C proteins sequence for: C.Csp231I family (21 sequences, upper part); C.PvuII family (65 sequences, bottom part). Helical structure, designated as seven white tubes, is derived from C.Csp231I crystal analysis (45). The regions of proteins responsible for activation of transcription and DNA recognition are inferred based on structural analysis of C.AhdI, C.Csp231I or mutational analysis other C.PvuII-like members. Grey boxes represent well conserved residues with the highest similarity regions between the two C protein families: C.PvuII and C.Csp231I. Logo was generated from software at http://weblogo.berkeley.edu. (**B**) Preparations of C-terminal His-tag fusion of WT C protein (3μg) and its variants: C-ARQmut (2.8μg) and C-SQEmut (2μg), resolved on a 10% acrylamide Tricine SDS gel and Coomasie Blue stained. Overproduction and purification were carried out as described in Materials and Methods section. (**C**) C.Csp231I and its variants binding to the P_C_ promoter/operator region (C-box) containing the two inverted repeats CTAAG-n_5_-CTTAG. A 576-bp target DNA fragment was prepared by PCR amplification, as well as its control with no C-box sequence (515-bp) containing a DNA fragment of comparable size but lucking the C-box (Supplementary Table S2). Each binding reaction was carried out with the same amount of DNA (100 nM) and increasing concentration of proteins: 0, 200, 500, 1000 and 2000 nM. Reactions were processed further as outlined in Materials and Methods and finally resolved on 6% native polyacrylamide gels. DNA was visualized by ethidium bromide staining. Open and filled arrows denote positions of unbound DNA and protein–DNA complexes, respectively. The comparable data were obtained with modified protocol (Supplementary Figure S3).

We next tested if the observed stimulation of transcription in presence of the C gene is due to C protein binding the inverted repeats (C-boxes) located upstream of the C gene. To date, most of the tested C proteins (C.PvuII-like) recognize two operators comprising nearly palindromic sequences of GACT-tat-AGTC, separated by a highly conserved central spacer with GT conserved at the center (36,68). C.Csp231I, which represents a new class of C proteins, was grouped based on the unusual sequence of the two inverted repeats, CTAAG-n_5_-CTTAG separated by an extended AT-rich 18-nt spacer, that showed no obvious similarity to the binding site of its homolog C.EcoO109I (33,45). Moreover, the C.Csp231I crystal structure revealed additional two helices at the C-terminus (H6 and H7; Figure 2A) that may play a role in dimer interface stabilization (45). Even with these differences the positional alignment of two large groups of C proteins (C.PvuII-like and C.Csp231I-like) still revealed highly similar short regions in the amino-acid sequence (Figure 2A, gray bars). With the exception of the regions linked to transcription activation and DNA recognition, no tests were performed to assign amino acid conservation to C protein features. The structural comparison of C.PvuII and λcI repressor (40) shows the common amino-acid region with Glu (E) residue as a vital contact to σ^70^ of RNA polymerase to achieve transcription activation (69). We also found a conserved region (G_14_LSQE_18_) in the C.Csp231I amino-acid sequence (Figure 2A), and generated a C.Csp231I protein variant in which S_16_Q_17_E_18_ of helix H2 were replaced; S16A; Q17A; E18A (designated C-SQEmut). We also constructed a C protein variant (A33G; R34E; Q37A) that is predicted to impair DNA binding (45) by inhibiting the engagement of H3 in DNA recognition (Figure 2A). We designated this C variant as C-ARQmut. Both variants, as well as the WT protein C.Csp231I, were expressed and purified to homogeneity (>95%) as C-terminal His-tag fusion proteins (Figure 2B). The predicted molecular mass of 12 046 Da for C.Csp231I-His matched the value estimated from SDS-PAGE. Interestingly, the C-WT fused to His-tag, unlike its changed variants, showed two bands on the SDS-PAGE gel that correspond to the monomer (more intense) and dimer even under the denaturing conditions used during the protein separation (Figure 2B). The identity of these two forms of C.Csp231I was confirmed by western blot (Supplementary Figure S2). This observation supports the presence of a strong dimerization interface for C.Csp231I that was previously predicted from the crystal structure (45).

Next, we tested whether or not the C.Csp231I-His and its variants bind to the DNA fragment containing the operator bearing both of the inverted repeats of C-box sequence *in vitro* (boxed sequences of O_L_ and O_R_ in Figure 1C, bottom). EMSA reactions were performed with the same amount of a 576-bp target DNA (100 nM) and increasing concentrations of the different C proteins (0–2000 nM) (Figure 2C). DNA of comparable size and with nonspecific sequence (no C-box) was used as a control in testing the specificity of the DNA-protein interaction. The specific shift in DNA-protein migration was observed only for C.Csp231IWT, but not for its variants: C-ARQmut or C-SQEmut (Figure 2C). The retarded complex was not distinct and some unbound DNA still remained. Different target DNAs (biotin-labeled 32-nt oligonucleotides) were also tried and they produced similar results (Supplementary Figure S3). Overall, these data indicate ARQ and SQE substitutions in C protein heavily disturb the interaction between the regulator and its operator within the inverted repeats.

In addition, we have noted a sequence between the MTase promoter hexamers (Figure 1C), which weakly resembles the single repeat of C-box recognized by C.Csp231I (Supplementary Figure S4). We performed EMSA reaction and did not obtain any retarded complex. C protein binding was not observed at least in tested conditions (Supplementary Figure S4).

### C.Csp231I positively and negatively regulates its own P_C_ promoter

**Figure 3.**
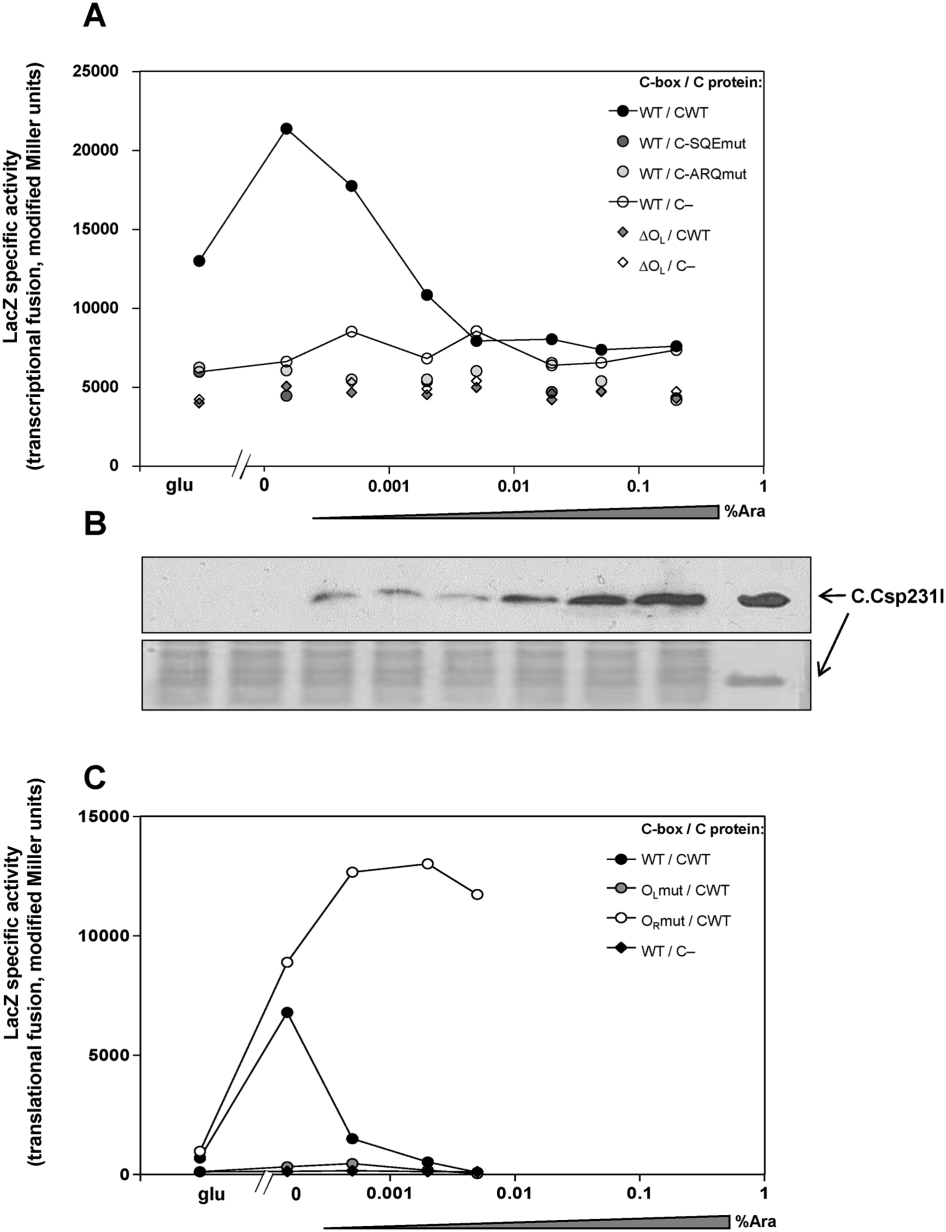
C.Csp231I is a transcriptional positive and negative autoregulator. Cells were grown in minimal media with 0.2% glucose (glu) or arabinose (ara) at indicated concentration from 0–0.2%. Cells contained two plasmids: one with WT promoter or its variant fused to reporter gene, second, compatible plasmid delivered WT C gene under inducible P_araBAD_ promoter, its C variant or C-absent vector. (**A**) Transcriptional profiles were measured as LacZ specific activity. Circles represent values for WT C gene promoter/ operator including two inverted repeats (O_L_ and O_R_) fused to *lacZ* reporter gene in p20 plasmid. Diamonds represent values for C gene promoter with O_L_ operator deleted and fused to *lacZ* reporter gene in p12 plasmid. As a source of C inducible expression following plasmids were used: pBAD-CWT; pBAD-sqe; pBAD-arq or pBAD33 vector as a C-negative control. For plot clarity, C.Csp231I effect on its own transcription is represented by connected points: black circles in C presence and open circles in C absence. (**B**) Crude extracts from cells showing WT C-dependent transcription profile indicated by black circle in panel A, were resolved by 10% acrylamide Tricine-SDS PAGE and analyzed by western blotting using the rabbit anti-C.Csp231I polyclonal antibodies to detect C protein level (visualized by chemiluminescence). Bottom part serves as a protein loading control stained by coomassie brilliant blue. (**C**) Effect of altered C-box on C-dependent transcription profile. Mutation within O_L_ or O_R_ were introduced (CTTAG→GTATC; pLex15-O_R_mut or pLex15-O_L_mut) and measured as translational LacZ activity in WT C protein presence (circles; pBAD-CWT) or C absence (diamond, pBAD33). Transcriptional (panel A) and translational (panel C) activity was measured as LacZ specific activity determined by linear regression of the slopes for the lines generated by plotting LacZ activity versus optical density of the culture (modified Miller units) (53). Error of each point was measured with R-squared value not less than 0.94. Note, LacZ activity is from a transcriptional fusion in panel A, and from a translational fusion in panel C.

In general, C proteins are dedicated transcription factors that activate and repress their own transcription. We examined if C.Csp231I also acts accordingly using a previously tested *in vivo* titration of C protein to verify (36). Our experimental system is based on two compatible plasmids, one of which produces the C protein in a controlled fashion (pBAD-CWT or its variants) while the second plasmid carries a reporter gene (*lacZ*) fused to the C.Csp231I operator/promoter sequence (p20), which is the target for C protein action. C expression is controlled by the *araBAD* promoter, which is repressed by glucose and induced by arabinose over a wide range (51). Thus, C protein level can vary from undetectably low to high levels as shown by western blotting (Figure 3B). The host bacteria were *E. coli* MC1061, which is deficient in *lac* and *ara*, but able to transport arabinose. Experiments were performed in minimal media and the effect of C protein dependent transcription from P_C_-*lacZ* was assessed by ONPG hydrolysis.

The results reveal that only WT C protein can activate the transcription from WT P_C_ promoter/operator yielding about a 4-fold increase compared to activity without C protein (Figure 3A). Interestingly, the peak of activity was achieved when no arabinose was added to the cells, and the C protein level was very minimal (undetectable by western blot) likely indicating expression leakage of P_araBAD_. Further increase in the C protein concentration were associated with progressive LacZ reduction, which reached a level seen without C protein at 0.002% arabinose. This demonstrates that, like several other C proteins, C.Csp231I acts as both an activator and a repressor of transcription. As a control, we used several combinations of C protein and C-box variants (WT and with O_L_ deleted; Figure 3A). These *in vivo* test of the C variants C-ARQmut or C-SQEmut confirmed our *in vitro* data (Figure 2C), as none of them could induce transcription activity above the no-C protein level, which stayed similar across the full range of arabinose concentrations.

To determine which operator (O_L_ and O_R_) upstream of the P_C_ promoter containing the two inverted repeats CTAAG-n_5_-CTTAG is associated with activation or repression (see Figure 1C, bottom sequence) we made separate mutations exchanging CTTAG for GTATC in each operator in pLex15, which has the C gene and its regulatory region fused to *lacZ*. The experiments were performed as above with a second plasmid carrying the WT C gene under control of P_araBAD_ or with the empty vector as a control. The results confirmed our prediction of a common regulatory pattern for C proteins. Mutation within O_L_ abolished the activation arm in the transcription profile yielding *lacZ* activity similar to that in the C-negative control (Figure 3C). In contrast, mutation within O_R_ leads to comparable activation to WT C-box, but results in loss of transcriptional repression in the presence of C protein (Figure 3C).

### C and REase genes share a bicistronic transcript

**Figure 4.**
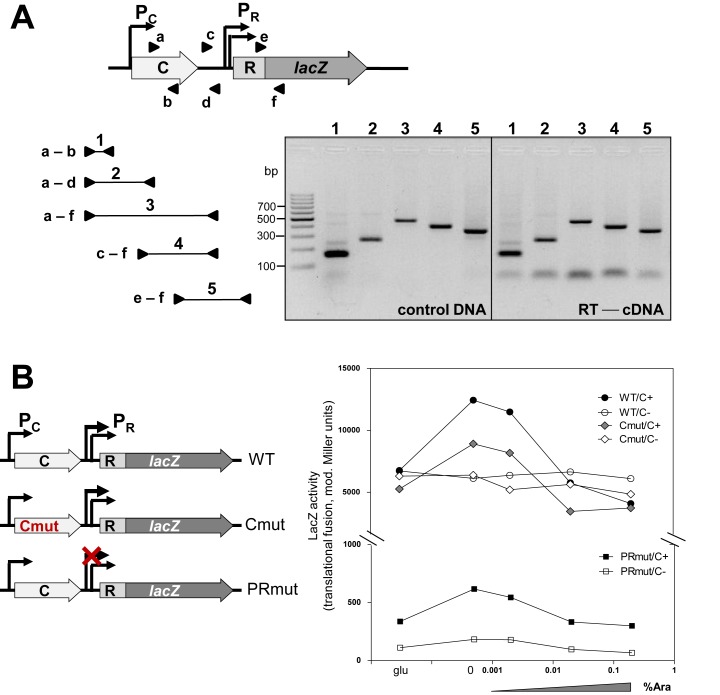
Transcripts initiated from P_C_ are bicistronic. (**A**) Schematic diagram of the C and R::*lacZ* genes in tested plasmid (not to scale). Triangles depict the primers (a–f) used in PCR reaction, where cDNA reversely transcribed from *E. coli* harboring pLexWT total RNA (right panel) or control pLexWT DNA (left panel) was used. The expected PCR products were: 1 (150bp); 2 (230bp); 3 (500bp); 4 (399 bp) and 5 (300 bp). (**B**) Transcription activity measured downstream of P_R_ promoter. The reporter *lacZ* gene was fused in-frame to REase gene generating a series of plasmids, which important features are indicated on the diagrams (red in left panel; not to scale). Briefly: Cmut indicates a substitution: A33G; R34E; Q37A in C protein; P_R1_ promoter was knocked-out as in Figure 1D. The transcription activity in context of a second plasmid carrying no C gene (pLex3B vector, white bars) or C gene (pBAD-CWT, dark bars) is presented in modified Miller units, as in Figure 3. Error of each point was measured with R-squared value not less than 0.95.

For all previously tested systems with C proteins (*e.g*. PvuII, EcoRV, AhdI, Esp1396I), a bicistronic C-REase mRNA initiating from the C gene promoter has been found. While our data for Csp231I indicate that the REase gene has its own promoter, it did not rule out possible readthrough transcription of the REase gene from the C promoter. To test whether this occurred we used reverse transcription PCR (RT-PCR) with primers encompassing the C/R region. PCR products were generated for each primer set located within the C or REase genes clearly indicating that a bicistronic transcript is made (Figure 4A).

Next, we wanted to test if P_C_ and P_R1,2_ contribute to REase expression equally or not. We used similar approach to the reporter assay discussed above (Figure 3), but in this case the reporter *lacZ* gene was inserted downstream to measure the REase expression level (Figure 4B). We quantified the REase transcriptional profile for a C protein gradient for WT (pLex-WT), for a variant defective in C protein binding to C-box (pLex-Cmut; as ARQ→GEA), and for another variant with an inactive P_R1_ (pLex-PRmut). P_R1_ knock out showed a drastic effect on REase expression (WT/C+ versus PRmut/C+) making it clear that the major effect on REase gene expression is via P_R1_ and not the C gene (Figure 4B). The mutation abolishing C binding to C-box resulted in slightly lower REase expression than WT (WT/C+ versus Cmut/C+) (Figure 4B). For the P_R1_ knock out we still see the effect of P_C_ driving the bicistronic mRNA as revealed by the peak of REase expression in presence of C protein at same concentration as for WT. We also noted that the delivery of WT C gene *in trans* for the variant disabled in C-box binding (Cmut/C+) did not fully restore the WT REase expression. Instead the REase expression was significantly increased at lower C concentration (Cmut/C+ versus Cmut/C-) (Figure 4B). Overall, this leads to the conclusion that C protein does control the REase expression, but the effect is only partial in magnitude making it unlike the other C-linked RM systems studied to date.

### C protein presence apparently is not essential for restriction endonuclease expression

**Figure 5.**
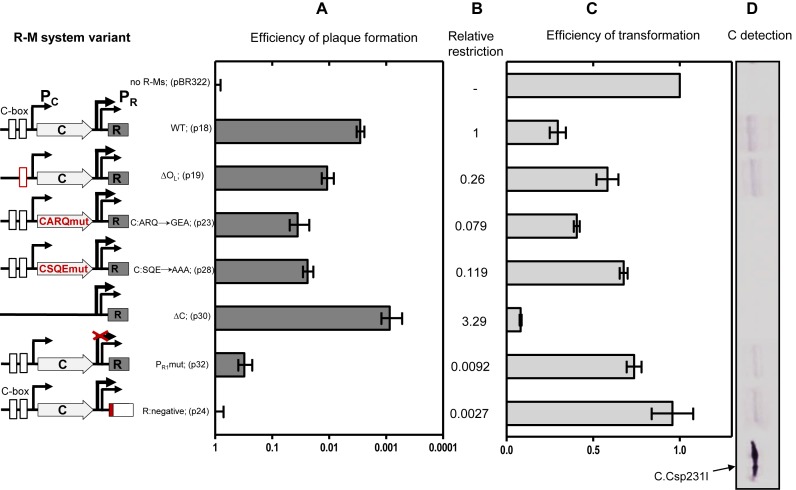
C protein is not essential for REase expression, but affects the R-M system transfer efficiency. Schematic diagram of the Csp231I R-M system WT and its variants (not in scale) are presented and data for particular panel A-D are adjusted to be read horizontally. The unchanged MTase gene is not shown. Changed elements are depicted in red color. Briefly: p18 (WT); p19 (deletion of O_L_ from C-box sequence); p23 (substitutions: A33G; R34E; Q37A in C protein); p28 (substitutions: S16A; Q17A; E18A in C protein); p30 (deletion of C gene and its upstream region including C-box and P_C_); p32 (mutation of -10 box of P_R1_, TTAAAT→CCCGGG); p24 (knock-out of REase gene, XhoI cut and Klenow filling) **(A)** Efficiency of plaque formation was assayed as plaque forming units on tested plasmid divided by plaque forming units on pBR322. The standard deviation from at least three experiments is shown. **(B)** Relative restriction is indicated as 1 for WT R-M system. **(C)** Efficiency of transformation is a relative number of transformants obtained from the same amount of plasmid DNAs carrying the indicated R-M system variants referred to vector pBR322 **(D)** Level of C protein in bacterial crude extracts was tested by western blot (horizontal bands) using the rabbit anti-C.Csp231I polyclonal antibodies and visualized by nitroblue tetrazolium (NBT) as the color development reagent. Culture samples were normalized by OD_600_. Loading control was prepared as one from Figure 3B; it is not shown for clarity. C.Csp231I protein is marked by arrow.

To further understand the role of C protein and other genetic elements in the modulation of Csp231I R-M system, the genetic variants already tested by LacZ translational fusion assays were implemented in the context of the entire R-M system (Figure 5, schematic maps). First, their biological effect was determined by plaque formation assay with use of λ*vir* bacteriophage, which carries six recognition sites for R.Csp231I. Changes to amino acid residues within the C protein that prevent binding to its C-box sequence (S_16_Q_17_E_18_→AAA, p23 and A_33_R_34_Q_37_→GEA, p28; Figure 5A) resulted in ∼8- and 11-fold decreases in restriction activity in comparison to the WT R-M system (p18) (Figure 5A). Similarly, a variant C-box sequence with deleted O_L_ (p19) also showed about 4-fold less restriction activity. The knockout of the major REase promoter (p32) had the most pronounced effect in reducing restriction activity at about 100 times less than WT, which is also in accord with our reporter assay (Figure 4B). Surprisingly, however, a deletion of the entire region upstream of P_R_ that included the entire C gene and its linked operator (p30) resulted in the highest phage restriction, that is, about three times that of the WT Csp231I R-M system (Figure 5AB). This *in vivo* observation apparently indicates that C protein is dispensable for the restriction activity of the Csp231I R-M system.

### The C gene-absent R-M system exhibits impaired establishment in new host cells

Typical C proteins act as ‘timing regulators’ to delay the appearance of toxic REase activity in new unprotected hosts during R-M system transfer (36). We next tested if the C protein is required for Csp231I R-M system establishment in a new host. The plasmids carrying the WT R-M system or its variants were introduced into cells, and the relative efficiency of transformation (EOT) was determined (Figure 5C). For some R-M systems high restriction activity contributes to poor establishment of the restriction plasmid due to the lethal effect on the acceptor cells. This is true of the p18 plasmid carrying the WT Csp231I R-M system (Figure 5C). Among its variants, only a REase-negative mutant (p24) showed a high transformation efficiency comparable to that of the control plasmid (pBR322). The wild-type and other variants had varied establishment defects that usually correlated with their cellular restriction activity level. The R-M system variants that displayed reduced efficiency of plaque formation (C gene mutation, p23 and p28 or C-box sequence deletion, p19; Figure 5B) also showed slightly better efficiency of transformation (1.5–2.5-fold) in comparison to WT. The most substantial impairment in R-M system establishment (4-fold) was observed for the variant lacking a C gene (p30), which is consistent with the observation that it yielded the highest REase activity (Figure 5C). The *E. coli* cells carrying R-M system variants were also examined for cellular C protein levels (Figure 5D). Only the variants with a C gene mutation preventing C protein binding (p23 and p28) were undetectable using the western-blot assay. The C gene deletion mutant (p30) yielded similar results. Other variants showed C protein level comparable to WT (Figure 5D). These data together demonstrate that the presence of the C gene is essential for improving the efficiency of Csp231I plasmid transfer, but not required for REase production.

### C protein presence improves R-M system host fitness

**Figure 6.**
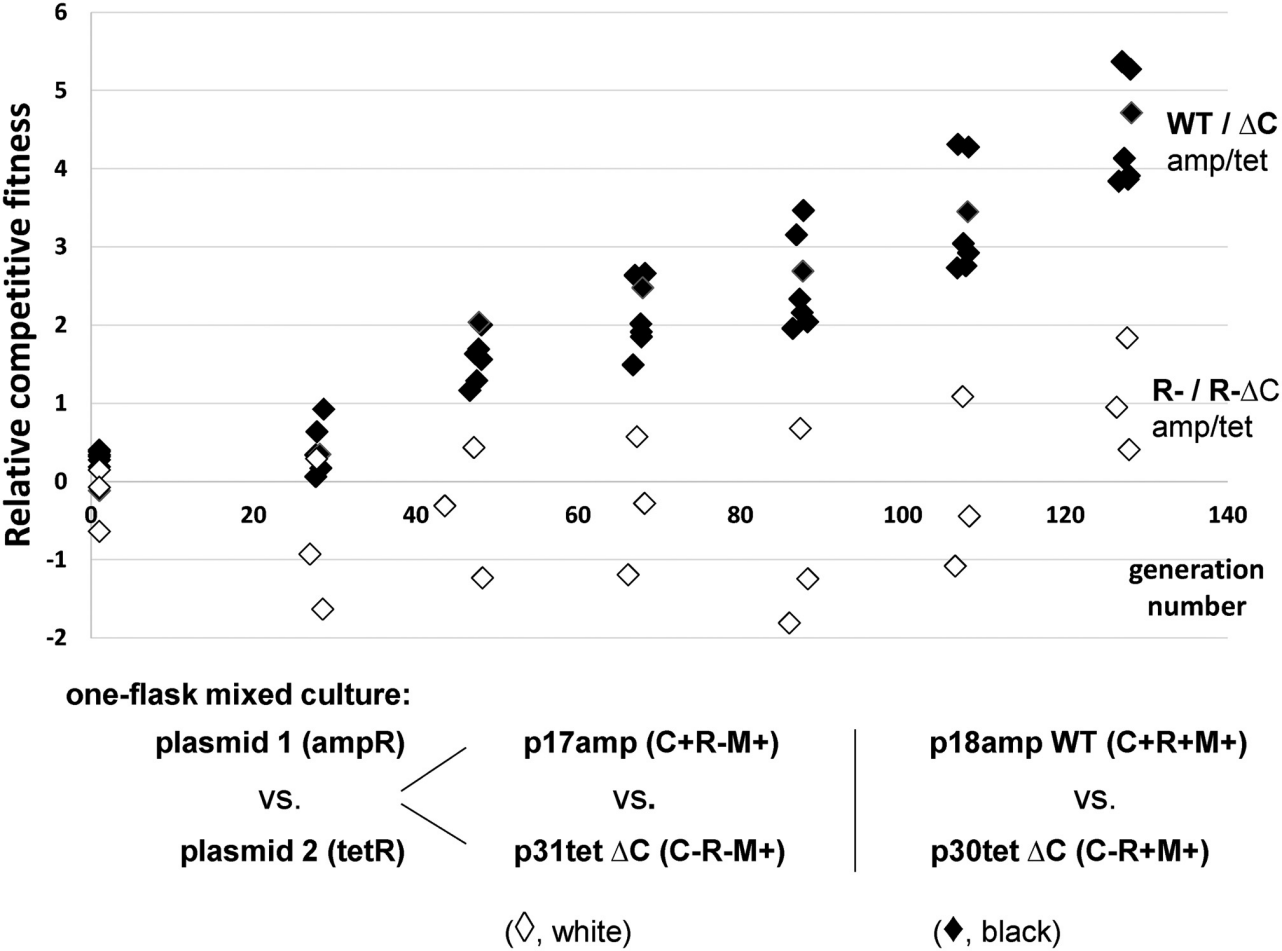
Cells with the WT R-M system have a fitness advantage over cells carrying the C-deleted variant. Mixed cultures were prepared by adding equal numbers of the two competing *E. coli* strains into medium without any antibiotic (Materials and Methods). Each type carried plasmid with a specific R-M system variant and its distinct antibiotic marker (tetracycline or ampicillin), as indicated below the diagram. One flask co-cultures were diluted every 24 generations into fresh medium and CFUs of competing cells were measured. Relative competitive fitness (W) was estimated individually for each mixed culture represented on diagram as a single symbol, calculated as *W* = log(CFU_amp_/CFU_tet_) and normalized to vector control (pBRamp versus pBRtet) (Materials and Methods). Black diamonds represents seven separate co-cultures, where WT R-M system in ampicillin resistant cells (p18amp) competed with C-deleted R-M system (p30tet) in tetracycline resistant cells. In control, parallel co-cultures, cells with plasmids with restriction-negative and modification-positive variants (p17amp *vs*. p31tet; white diamonds) were used.

We also questioned whether the WT R-M system and its C-deleted variant, which both have comparable restriction activity (p18 versus p30, Figure 5) would show any differences in viability or fitness. We challenged the strains in a direct one-flask competition assay by mixing equal numbers of cells carrying a WT R-M system or its C-deleted variant on a plasmid. Different antibiotic resistance genes served as the cell markers: p18ampWT (C+R+M+) *vs*. p30tetΔC (C-R+M+) and in parallel controlled flasks restriction-negative R-M systems: p17amp (C+R-M+) versus p31tet (C-R-M+) were tested in identical conditions. Vector control (pBRamp versus pBRtet) served to normalize the experiments in case the antibiotics-resistance expression conferred some fitness advantage. We started the cultures and let them grow for about 130 generations in antibiotic-free minimal medium. Sub-cultures were created every 24 generations, and the number of colony-forming units were counted (Figure 6). The results show a significant gain in relative fitness advantage for WT R-M system over its C-deleted variant (WT/ΔC, Figure 6) in which REase expression relies only on the separate tandem promoters. We also confirmed the competed cells maintained their high restrictive-phenotypes for the entire course of the experiment. For controls with restriction-negative R-M systems we found the difference in the relative fitness to be insubstantial, particularly, for those measurements taken within 100 generations (R- CWT versus R- ΔC, Figure 6).

## DISCUSSION

We report here data on the regulation of expression for Csp231I R-M system with a C protein of a new class. According to the Sorokin classification C proteins fall into groups based on their distinct motifs in DNA sequence binding, which for C.Csp231I and its prototype C.EcoO109I (motif 8 – ACTAAGGA-T-TnCTTAGT) is a small fraction of the entire C protein number (43,44). C.Csp231I and C.EcoO109I share about 70% identity in amino acid sequence and are significantly larger proteins at about 11 kDa (59). They are also different structurally from other studied C proteins with motifs 1–6 (∼8–9 kDa). They possess two extra helices at the carboxyl terminus that may play a role in dimer formation as inferred from the solved C.Csp231I crystal structure (45,46). However, C.Eco0109I appears to operate by a mode of regulation typical for the majority of C proteins as its C gene inactivation leads to loss of REase production (33) and this is unlike the regulatory mode reported here for C.Csp231I.

### Similarity in action of C.Csp231I to other R-M system control proteins

Despite the structural distinction of C.Csp231I and its C-box some essential features of transcriptional auto-control of other studied C proteins are maintained. We mapped the C gene promoter and determined its –10 and –35 hexamers. The produced transcript appears to be leaderless (70), which is a common property of operons for R-M systems associated with C proteins belonging to the C.PvuII and C.EcoRV families (27,35,40). The key element in C gene control over REase expression seems to be the shared mRNA present in majority of C-linked R-M systems as well as toxin-antitoxin systems associated with analogous transcription factors (10,71,72). We also detected bicistronic mRNAs for the C and REase genes (Figure 4AB). The C-box of C.Csp231I is formed by two palindromic sequences CTAAG-n_5_-CTTAG separated by 18nt with A/T rich spacers (Figure 1C, bottom sequence). C binding to these sequences is likely to bend DNA tightly, as shown for other C proteins (33,37,68). In addition, C.Csp231I also uses an autoregulatory feed-back loop to switch the transcription profile from activation to repression. C proteins binding to the O_L_ site as a dimer (45) directly contact RNA polymerase σ^70^ subunits (region 4, R588) via a short highly conserved amino acid sequence (SQE) found in most C proteins (Figure 2A) and phage repressors (40,73). This contact results in transcription activation as seen for λCI (69,74,75) and based on modeling the interaction between σ^70^ and in ternary complex with C.AhdI dimer and DNA (73). Indeed, a replacement of the key S_16_Q_17_E_18_ residues of C.Csp231I led to loss of transcription activation as tested *in vivo* (Figure 3A). We expected that C binding for such variant would not be disturbed *in vitro*; however, binding of C-SQEmut variant to its WT C-box was not observed during our tests (Figure 2C). The analyzed C dimer – DNA crystal structure indicates the Q_17_ residues are located close enough (<3 Å) to interact with the DNA phosphate backbone (Supplementary Figure S5; (46)). In addition, the S_16_ substitution by A may destabilize the H2 helix as the serine residue exerts more propensity toward N-capping than the alanine residue (76). Overall, such an effect, through indirect readout, may subtly change the DNA's ability to achieve the required conformation as evidenced for some bacteriophage repressors (77,78). In a similar manner we tested the A_33_R_34_Q_37_ replacement in C protein that is predicted to weaken DNA binding (45), and it showed comparable results to the S_16_Q_17_E_18_ variant including loss of DNA binding *in vitro* (Figure 2C), inability to activate C-mediated activation of transcription (Figure 3A), decreased phage restriction *in vivo* (Figure 5AB) and diminished level of C protein in cells (Figure 5D). We also showed that as with other C proteins the repression step in transcription is associated with inverted repeats located closer to the C transcription start (O_R_), which are overlapped by the -35 hexamer of P_C_ (Figure 3C). Saturation of O_L_ and O_R_ after C protein accumulation by a presumable C tetramer (46) leads to transcriptional autorepression. A similar switch in transcription (activation *vs*. repression) is linked to the highly cooperative binding of two C proteins dimers, and was identified for C.AhdI, C.PvuII, C.Esp1396I (36,38,79). Binding is not cooperative for C.Csp231I and C.EcoRV (27,46). Interestingly, the C.Csp231I activation achieved the highest values at C protein concentration below the detection threshold of our assay (Figure 3) suggesting that in this R-M system C protein regulation has been tuned to operate at low copy/chromosomal levels.

### Novel elements in R-M system regulation with C protein of a new class

The Csp231I R-M system contains collinearly oriented C and REase genes that are separated by 152 nt. We found that there are two functional promoters, P_R1_ and P_R2_, for the REase gene in the region between the C and REase genes (Figure 1BC). This independent transcription is novel among the known C-associated R-M systems. A constitutive promoter for the REase was found in the Kpn2I R-M system, but its C gene is located divergently at some distance from the REase gene (39). The tandem promoters are the major source of REase transcription with only minor contribution of bicistronic mRNA driven by C gene promoter (Figure 4B). Our *in vivo* data show that the C gene effect on REase production is positive, but it is not large, which is unlike C.PvuII, where inactivation of C leads to complete loss of restriction activity (26). In this system abolishing C binding to DNA (SQE and ARQ variants) as tested *in vitro*, still maintains a restrictive phenotype (Figure 2C). The restriction activity was tested in plaque formation efficiency assays using λ*vir* phage (Figure 5BC), and was found to be decreased by 8–12-fold. This C protein positive effect is due to production of a shared C/R mRNA that is regulated by P_C_, and can be elevated in response C protein binding to its C-box (feed-back response) (Figure 4B, pLex-WT *vs*. pLex-Cmut). Deletion of the entire C gene resulted in a 3-fold increase in phage restriction (Figure 5AB) and severely affected the host cell ability to compete with other restriction-positive cells (Figure 6). The reduction of REase expression in presence of the C gene may be the effect of transcription readthrough, which prevents RNA polymerase from binding to REase promoters. Hence REase expression is slightly attenuated in presence of C gene. Such modulation of toxic expression apparently is needed, otherwise leads to elevated toxicity and its host fitness compromised.

In this work, we report the first case of a C protein-associated R-M system in which the C gene positively affects REase production, but is not required for its expression. In general, the C protein control operates at two important stages: R-M system maintenance and establishment in a new cell (14). The maintenance stage was tested by the stability assay separately for WT and C-deleted R-M system and appeared to be comparable (data not shown). However, the direct competition fitness assay showed the cells carrying the C-deleted R-M system, in which REase expression relies on the separate tandem promoters, were outcompeted by the cells with WT R-M system. This result clearly indicates the C protein improves its host cell fitness, and is vital for R-M system propagation. Loss of fitness for C-deleted R-M system cells might occur due to autorestriction of its host genome when the R-M system activities are not finely balanced leading eventually to cell death and a heavily perturbed the mixed cell population ratio (8,9,80,81). We also tested whether the C.Csp231I may be essential when R-M system needs to enter the unprotected new cell. We demonstrated that the presence of the C gene significantly helps the R-M system during entry to a new cell (Figure 5C). The higher restriction activity the more lethal the effect appeared during the R-M system transfer. It remains to be determined how newly identified aspects of regulation mode for C.Csp231I and its R-M system exert the delay in expression of toxic restriction endonuclease in the cell. It seems such delay mechanism via C protein partial control, may not be sufficient for this particular system, and other patterns of temporal control may operate instead, e.g. by stimulation of MTase expression. The presence of the possible multi-layered complexity is not surprising as R-M systems and other toxin-antitoxin modules must be controlled to keep the counter-balancing amounts and timing properties to avoid lethality (10,12).

## SUPPLEMENTARY DATA

Supplementary Data are available at NAR Online.

SUPPLEMENTARY DATA
