# Supplementary material for: Natural C-independent expression of restriction endonuclease in a C protein-associated restriction-modification system
Source: Nucleic Acids Res. 2015 Dec 9;44(6):2646–60. doi: 10.1093/nar/gkv1331 (PMC4824078; doi:10.1093/nar/gkv1331)
Supplement: SUPPLEMENTARY DATA [file supp_44_6_2646__index.html]

Natural C-independent expression of restriction endonuclease in a C protein-associated restriction-modification system — SUPPLEMENTARY DATA 

# Natural C-independent expression of restriction endonuclease in a C protein-associated restriction-modification system

## SUPPLEMENTARY DATA

- SUPPLEMENTARY DATA
