# Supplementary material for: Natural C-independent expression of restriction endonuclease in a C protein-associated restriction-modification system
Source: Nucleic Acids Res. 2015 Dec 9;44(6):2646–60. doi: 10.1093/nar/gkv1331 (PMC4824078; doi:10.1093/nar/gkv1331)
Supplement: SUPPLEMENTARY DATA [file supp_gkv1331_nar-03125-h-2015-File008.doc]

**Supplementary Data for:**

**Natural C-independent restriction activity in a C protein associated**

**restriction-modification system.**

Running title: *Csp231I R-M system regulation*

Key words: transcription regulation/ promoter / operator / DNA-protein interaction/ restriction-modification systems/

**Monika Rezulak, Izabela Borsuk and Iwona Mruk***

^1^Department of Microbiology, University of Gdansk, Wita Stwosza 59, Gdansk, 80-308, Poland

*Corresponding author

Abbreviations: *E. coli*, *Escherichia coli*; REase, restriction endonuclease; MTase, DNA methyltransferase; R-M, restriction–modification;

## Address for correspondence:

Dr Iwona Mruk

Department of Microbiology, University of Gdansk,

Wita Stwosza 59, 80-308 Gdansk, Poland

Tel: (58) 5236071

e-mail: shamrock127@hotmail.com

**Table S1**. Plasmids used in this study.

| **name** | **relevant features/cloning** | **references** |
| --- | --- | --- |
| pLex3B | vector for testing gene translational fusion to *lacZ* reporter, pBR322 ori, AmpR | (1) |
| pLexWT | as pLex3B, but WT fragment encompassing C gene promoter, C gene, REase promoter, REase gene 5’ is fused in frame to *lacZ* gene /  PCR amplified fragment (primers lacFus1 and lacFus2) was digested with XhoI and HindIII and cloned into linearized vector pLex3B with XhoI i HindIII | This study |
| pLex-Cmut | as pLexWT, but triple amino acid residue of C.Csp231I substituted as followed A(GCA)33®G(GGA); R(AGG)34®E(GAG); Q(CAG)37®A(GCG) /  pLexWT mutagenized with primers arq1 and arq2 | This study |
| pLex14 | as pLexWT, but P_R1_  promoter of REase gene knocked-out by replacing its -10 box TTAAAT into CCCGGG /  pLexWT mutagenized with primers PRmut3 and PRmut4 | This study |
| pLexP_R_WT | as pLexWT, but only WT fragment of REase gene 5’ is fused in frame to *lacZ* gene, no C gene and its operator is present /  pLexWT was digested with Xho and SphI, T4 DNA Polymerase treated and autoligated | This study |
| pLexP_R_mut | pLexP_R_WT, but promoter of REase gene is knocked-out as in pLex14 /  pLex14 mutagenized with primers PRmut3 and PRmut4 | This study |
| pLex15 | as pLex3B, but fragment of WT C-box and C gene 5’ is fused in frame to *lacZ* gene /  PCR amplified fragment (primers lacFus1 and lacFus5) was digested with XhoI and HindIII and cloned into linearized vector pLex3B with XhoI i HindIII | This study |
| pLex15-O_R_mut | as pLex15, but O_R_ of C-box upstream of C gene is mutated CTTAG→GTATC /  pLex15 mutagenized with primers ORmutF and ORmutR | This study |
| pLex15-O_L_mut | as pLex15, but O_L_ of C-box upstream of C gene is mutated CTTAG→GTATC /  pLex15 mutagenized with primers OLmutF and OLmutR | This study |
| pRS415 | vector for testing gene trancriptional fusion to *lacZ* reporter, pBR322 ori, AmpR | (2) |
| pM4 | as pRS415, but with upstream region of MTase gene  fused to *lacZ* gene /  PCR amplified fragment (primers C32 and C33) was digested with BamHI and EcoRI and cloned into linearized vector pRS415 with same enzymes | This study |
| pR3 | as pRS415, but with upstream region of REase gene  fused to *lacZ* gene /  PCR amplified fragment (primers C23 and C24) was digested with BamHI and EcoRI and cloned into linearized vector pRS415 with same enzymes | This study |
| p20 | as pRS415, but with WT upstream region of C gene  fused to *lacZ* gene /  PCR amplified fragment (primers C20 and C16) was digested with BamHI and EcoRI and cloned into linearized vector pRS415 with same enzymes | This study |
| p12 | as pRS415, but with upstream region of REase gene  fused to *lacZ* gene /  PCR amplified fragment (primers C12 and C16) was digested with EcoRI and cloned into linearized vector pRS415 with same enzyme | This study |
| pBAD24 | arabinose inducible *araBAD* promoter (P_BAD_), *araC*, pBR322 ori, AmpR | (3) |
| pBAD33 | arabinose inducible *araBAD* promoter (P_BAD_), *araC*, pACYC ori, CmR | (3) |
| pBAD-CWT9 | as pBAD24, but C gene under of P_BAD_ promoter /  C gene was amplified by PCR (primers PNK treated BAD7 and C7) and XbaI digested, cloned into pBAD24 digested with NcoI, Klenow filled and digested with XbaI | This study |
| pBAD-CWT | as pBAD33, but C gene under of P_BAD_ promoter /  insert with C gene was prepared by digestion of HindIII and NheI fragment of pBAD-CWT9 and cloned into pBAD33 digested with same restriction enzymes | This study |
| pBAD-arq | as pBAD-CWT, but C gene mutated as in pLex-arq /  pBAD-CWT mutagenized with primers arq1 and arq2 | This study |
| pBAD-sqe | as pBAD-CWT, triple amino acid residue of C.Csp231I substituted as followed S(TCA)16®A(GCA); Q(CAG)17®A(GCG); E(GAA)18®A(GCA) /  pBAD-CWT mutagenized with primers sqe1 and sqe2 | This study |
| pET28(+) | IPTG inducible T7 promoter, pBR ori, KmR | Novagen |
| pET-CWT | as pET28(+), but C gene under T7 promoter, which  produced a carboxylic-terminally His5-tagged C protein /  C gene was amplified by PCR (primers CNco and CRev), digested with NcoI and EcoRI and cloned into  pET28 digested with same enzymes | This study |
| pET-Csqe | as pET-CWT, but C gene mutated as pBAD-sqe / pET-CWT mutagenized with primers sqe1 and sqe2 | This study |
| pET-Carq | as pET-CWT, but C gene mutated as pBAD-arq /  pET-CWT mutagenized with primers arq1 and arq2 | This study |
| pEcoVIIIM | EcoVIII MTase gene under its natural promoter in pACYC177 vector, KmR  EcoVIII and Csp231I MTases have the same specificity of methylation | (4,5) |
| p18 | same as pCsp231I plasmid, carrying entire WT Csp231I R-M system, pBR ori, TetR | (4) |
| p19 | as p18, but C gene operator has deletion of O_L_ /  PCR amplified fragment (C12 and C7) digested with EcoRI was cloned into p18 linearized with EcoRI | This study |
| p23 | as p18, but C gene mutated as pLex-arq /  p18 mutagenized with primers arq1 and arq2 | This study |
| p28 | as p18, but C gene mutated as pBADsqe /  p18 mutagenized with primers sqe1 and sqe2 | This study |
| p30 | as p18, but Csp231I R-M system is devoid of C gene and its operator, but REase and MTase genes are intact /  PCR amplified fragment (C23 and C7) digested with EcoRI was cloned into p18 linearized with EcoRI | This study |
| p32 | as p18, but promoter of REase (P_R1_) is knocked-out as in pLex14 /  p18 mutagenized with primers PRmut3 and PRmut4 | This study |
| p24 | as p18, but REase gene is inactive  p18 was linearized with XhoI (within REase gene), Klenow filled and autoligated | This study |
| p18amp | as p18, but a tetracycline resistance gene disrupted by a insertion of a *bla* gene (PCR amplified, primers bla1 and bla3), Csp231I R-M WT | This study |
| p30tet | identical as p30, Csp231I R-M with C gene deleted | This study |
| p17amp | as p18amp, but REase gene is inactive  p18 was linearized with XhoI (within REase gene), Klenow filled and autoligated, as in p24 | This study |
| p31tet | as p30tet, but but REase gene is inactive  p18 was linearized with XhoI (within REase gene), Klenow filled and autoligated, as in p24 | This study |
| pBRamp | as pBR322, but 29bp DNA insert was clone into BamHI to disrupt the tetracycline resistance gene | This study |
| pBRtet | as pBR322, but 22bp DNA insert was clone into PstI to disrupt the ampicillin resistance gene | This study |

**Table S2**. Used oligonucleotides or DNA substrates

| **Experiment** | **Primers** | **Sequence (5’→3’)** |
| --- | --- | --- |
| translational  and transcriptional  fusion to *lacZ* gene | lacFus1 | TCCCTCGAGTAATAAAAGCAACGAGG |
|  | lacFus2 | TGAAAAGCTTTAATTCAGAAGAATCTAAATCAC |
|  | lacFus5 | GTCAAGCTT TCTTCTTATTAACATAAAAACCTC |
| mutagenesis | sqe1 | CGGGCTGGAATTGCAGCGGCAAAACTTGGAGTGC |
|  | sqe2 | CCAGCACTCCAAGTTTTGCCGCTGCAATTCCAGC |
|  | arq1 | GATGAGGCTTCGGCTAGTGGAGAGATGAATGCGTACGAAAAGGGTAAGC |
|  | arq2 | GCTTACCCTTTTCGTACGCATTCATCTCTCCACTAGCCGAAGCCTCATC |
|  | PRmut3 | GTTAAAAAATGGCCCGGGATGCAAATAATATAATCATTGG |
|  | PRmut4 | CCAATGATTATATTATTTGCATCCCGGGCCATTTTTTAAC |
|  | ORmutF | CTAAGAAAATGTATCCAAAAGTGTATGGCTGAG |
|  | ORmutR | CTCAGCCATACACTTTTGGATACATTTTCTTAG |
|  | OLmutF | CACACTAAGGAAAAGTATCTAAAATTGC |
|  | OLmutR | GCAATTTTAGATACTTTTCCTTAGTGTG |
| EMSA oligonucleoti-des | FBIOT1 | Biotin-TAATCACACTAAGGAAAACTTAGTAAAATTGC  (inverted repeats underlined) |
|  | BIOT1 | GCAATTTTACTAAGTTTTCCTTAGTGTGATTA  (inverted repeats underlined) |
| EMSA substrates | C20-C24  (specific) | AATGAATTCTAAAAGCAACGAGGAGGTCTATAAAAGGCCAAAGTAATCACACTAAGGAAAACTTAGTAAAATTGCTTTTTTAAACTAAGAAAATCTTAGCAAAAGTGTATGGCTGAGGTTTTTATGTTAATAAGAAGATTAAAAGACGCACGTCTTCGGGCTGGAATTTCACAGGAAAAACTTGGAGTGCTGGCGGGTATTGATGAGGCTTCGGCTAGTGCAAGGATGAATCAGTACGAAAAGGGTAAGCATGCGCCTGATTTTGAAATGGCCAACAGATTAGCAAAAGTGCTAAAAATTCCCGTTTCATACCTGTATACACCAGAGGATGATTTAGCCCAAATAATTTTAACATGGAATGAATTAAATGAACAAGAAAGAAAAAGAATCAATTTTTATATCAGAAAAAAAGCTAAATGAAACTATAATCTAGACACTATAGTTAAAAAATGGTTAAATATGCAAATAATATAATCATTGGAAGGAATCATGATGAAAGTTGTAAAAATGGTGAAGATCCCGCCTTTAGCTCAAGATTGTGATTTAGATTCTTCTGAATTATCAGAATTCACTCCAG  (inverted repeats underlined) |
|  | C2-C30  (non-specific) | CCCTTAACCATTCAGATGCCCACGTTTGACACCATTGGTAGTATTCAGCTGGGATTTTTTTATCTGCATCTGACCATCCATTGATTGGTTTTCCCCTGCGCTTAAAAACATTTCCAGCTTGTTTTTGAGCCGGACTATTTCCTAAATATGCTGTATTTGTATTTTTATGTAAAACATCCCAATCGTCAGCACCTATGCCATAAGGAATATCACTTAGAATAAGGTCTATGCTATTATCTTCTAACTTTTTTAGTTCGCTAATAGAATCTGCATTTATTAATAACTGCTTTCGCATTTCATTTGAATCCTATAAATGTTTCTATATATAATAACATATAATTAGGTAATAGTAAAATTCATCAAAAAATGCACTTTGTAATAGTTTAAGATTATAGATGTGTGCTCATTTACTTATGATTTAATTGTGTTAAATAAAACTTTTGCTAGAGAAATTATTTCGGTTGAATTTTAATTCGATTAATATGGCTCACAATATTCATCATAATGCTCTACGAT |
| Cloning | C20 | AATGAATTCTAAAAGCAACGAGG |
|  | C2 | CCCTTAACCATTCAGATG |
|  | C7 | ATGATCACTAAACCAACG |
|  | BAD7 | TTAATAAGAAGATTAAAAGACGCACG |
|  | C12 | AAGGAATTCTTAGCAAAAGTG |
|  | bla1 | AGTACAGCTGAGTAAACTTGGTCTGACAG |
|  | bla3 | ACGTCAGCTGGCACTTTTCGGGGAA |
|  | C23 | AAAGAATTCATTTTTATATCAG |
|  | C24 | CTGGATCCAATTCTGATAATTCAG |
|  | C30 | GCATTTCGAAATACTACTTATAACACTC |
|  | C32 | AATGAATTCTCTAGCAAAAGTTTATTTAACA |
|  | C33 | GGATCCGCATTTATTAATAAC |
| RT-PCR | a | TGCTGGCGGGTATTGATG |
|  | b | GGCTAAATCATCCTCTGGTGTAT |
|  | c | GCTAAATGAAACTATAATCTAGACACTATAGT |
|  | d | ACTATAGTGTCTAGATTATAGTTTCATTTAGC |
|  | e | AATCATTGGAAGGAATCATGATGA |
|  | f | CAAACTCCCCTGCTGCTGT |
|  | g | TTAATAAGAAGATTAAAAGACGCACG |
|  | h | GGCTAAATCATCCTCTGGTGTAT |
|  | i | AAGGAATTCTTAGCAAAAGTG |
| C protein  over-production | C-rev | ATGAATTCTTAGTGGTGGTGGTGGTGTTTAGCTTTTTTTCTGATATAAAAATTG |
|  | CNco | GGTTTCCATGGTAATAAGAAGATTAAAAG |
| promoter mapping | EXMET | CACTTAGAATAAGGTCTATGCT |
|  | EXRES | ATCTAAATCACAATCTTGAGC |
|  | C16 | GTGAGGATCCAGCCCGAAGACG |
|  | | |

ł


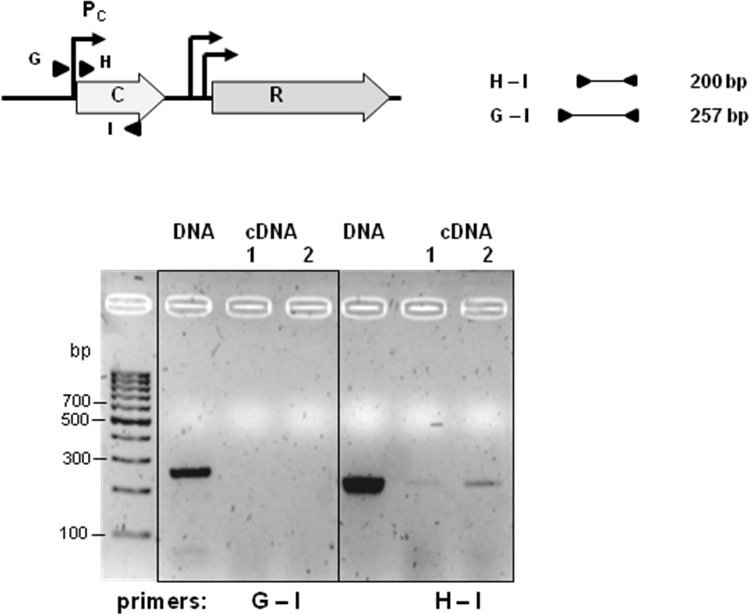


**Figure S1.** Confirmation of correct P_C_ promoter mapping. Schematic diagram of the C and REase genes location in Csp231I RM system (not to scale). Triangles depict the primers (G, H, I) used in PCR reaction. Control PCR reaction on DNA template was performed. Lane 1 – PCR on template of cDNA reversely transcribed from total RNA isolated from *E. coli* harboring plasmid with WT R-M system; p18; Lane 2- same reaction made on template of *E. coli* with plasmid p23 with C gene variant (ARQmut). Expected PCR product length is 200 bp for H–I primers and 257bp for G–I primers. Primers’ sequence is given in Table S2. Reverse transcription experiments were performed as indicated in Method section of the main manuscript.


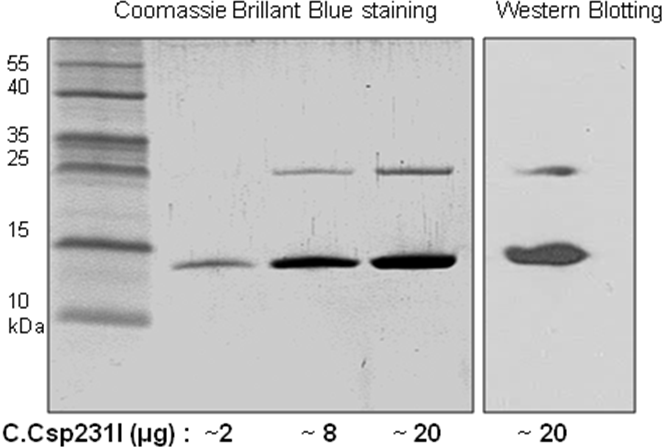


**Figure S2.** Confirmation of C.Csp231I identity by western blotting.

Preparations of C-terminal His-tag fusion of WT C protein at indicated amount were resolved on a 10% acrylamide Tricine SDS gel and Coomasie Brillant Blue stained. Overproduction, purification and western blot analysis were carried out as described in Materials and Methods section.


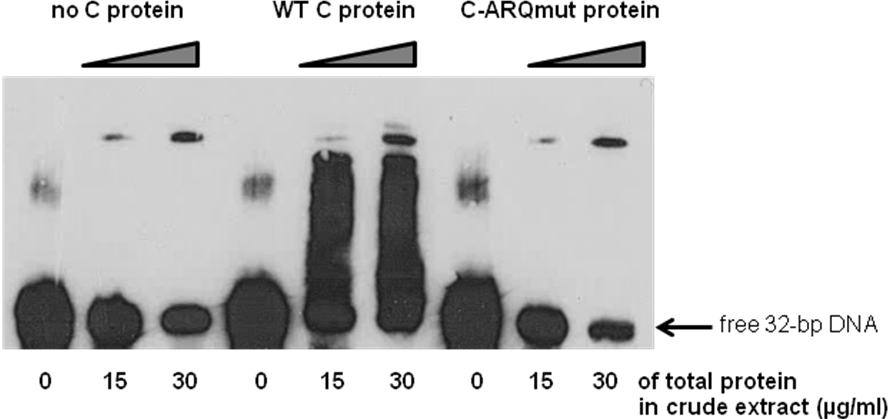


**Figure S3.** Electrophoretic mobility shift (EMSA) assays using bacterial crude extracts to show the specific C protein – DNA interaction.

**Crude extract preparation**

Bacteria were grown at 37°C in 10 ml of LB medium with appropriate antibiotic until OD_600_ ~0.5. Expression of C protein was induced in cells carrying plasmids: pBAD-CWT; pBADarq or pBAD33 with arabinose and incubation continued for 3 hours. Cells were harvested at 1600 *g* for 10 min. The pellet was resuspended in 1 ml of buffer comprising 20 mM Tris–HCl pH 8 and 50 mM NaCl. The cells were sonicated and then centrifuged for 30 min at 15500 *g* at 4^o^C. Supernatant was dialysed against the same buffer containing 10 % glycerol. The samples were aliquoted and stored at -20^o^C.

#### Electrophoretic mobility shift assays (EMSA)

5’-biotinylated, double-stranded fragments that included the C-box regulatory regions were used in EMSA. Reactions containing 100 nM of biotin–labeled DNA and bacterial crude extract at concentrations of 0, 15 and 30 µg/ml total protein were assembled in binding buffer (500 mM Tris-HCl pH 8, 10 MgCl_2_, 1 µg of competitor DNA in final volumes of 20 µl and incubated for 20 min at 22°C. Then samples were electrophoresed on 12 % native polyacrylamide gels in 0.5x TBE buffer for 120 min at 100V. DNA was transferred by electroblotting to positively–charged nylon membrane (Biosciences), and the transferred DNA fragments were immobilized onto the membrane by ultraviolet cross-linking. Detection of the biotin–labeled DNA was performed using the LightShift^TM^ chemiluminescent EMSA kit (Pierce) as recommended by manufacturer.


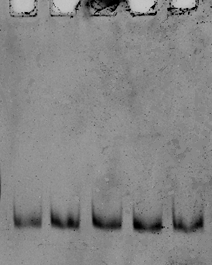

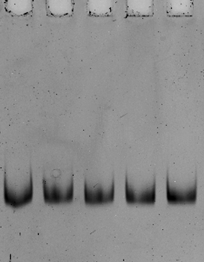


non-specific DNA

P_M_ promoter DNA

CWT

CWT

TAC**CTAA**TTATATG**TTA**TTA P_M_ DNA

ACA**CTAAG**GAAAA**CTTAG**TA O_L_

AAA**CTAAG**AAAAT**CTTAG**CA O_R_

**Figure S4.** C protein does not bind a DNA fragment containing the promoter for MTase *in vitro*, which shows some low level of identity (red) to C-box sequence (black bold).

We have noted a sequence amid of MTase promoter (Figure 1C; between -35 and -10 hexamers), which partially matches (denoted in red) one repeat of C-box (marked in bold), which is recognized by C.Csp231I (Figure 2C).

Due to some *in vivo* C protein effect on MTase promoter (Figure 1B), we tested if such effect could be explained by C binding to DNA within P_M_.

As substrate to EMSA reaction, PCR-amplified DNA fragment (C30-C33 primers, 250bp) that included the promoter of M.Csp231I was used.

In addition, a similar size irrelevant DNA fragment was prepared to serve as a negative control (PCR-amplified 320bp; C24-C25 primers).

Assay was performed as outlined in Material and Methods and Figure 2C of the main manuscript.

**Figure S5.** C protein dimer interaction with O_R_ operator (PDB, 4JCY; (6)). (DNA sequence below).


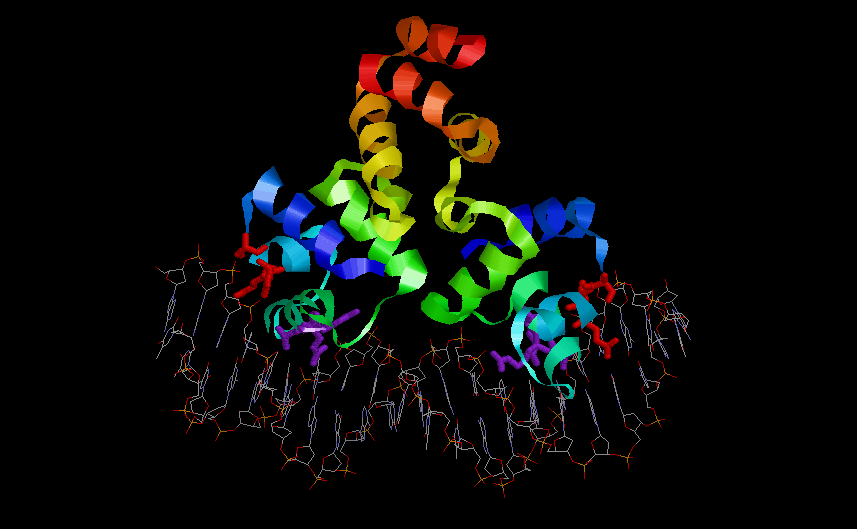


5’-AAACTAAGAAAATCTTAGCAA-3’

3’-TTTGATTCTTTTAGAATCGTT-5’

C protein 7 helices are in the same color in each monomer in the rainbow spectrum from blue (N-terminus) to red (C-terminus). Predicted amino acid residues engaged in DNA binding A_33_R_34_Q_37_ are labeled in magenta (7). The analyzed C dimer – DNA crystal structure indicates the Q_17_ residues (S_16_Q_17_E_18_  labeled in red) are located close enough to interact with DNA sugar-phosphate backbone.

**Supplemental References**

1. Diederich, L., Roth, A. and Messer, W. (1994) A versatile plasmid vector system for the regulated expression of genes in Escherichia coli. *Biotechniques*, **16**, 916-923.

2. Simons, R.W., Houman, F. and Kleckner, N. (1987) Improved single and multicopy lac-based cloning vectors for protein and operon fusions. *Gene*, **53**, 85-96.

3. Guzman, L.M., Belin, D., Carson, M.J. and Beckwith, J. (1995) Tight regulation, modulation, and high-level expression by vectors containing the arabinose PBAD promoter. *J Bacteriol*, **177**, 4121-4130.

4. Mruk, I. and Kaczorowski, T. (2007) A rapid and efficient method for cloning genes of type II restriction-modification systems by use of a killer plasmid. *Appl Environ Microbiol*, **73**, 4286-4293.

5. Mruk, I. and Kaczorowski, T. (2003) Genetic organization and molecular analysis of the EcoVIII restriction-modification system of *Escherichia coli* E1585-68 and its comparison with isospecific homologs. *Appl Environ Microbiol*, **69**, 2638-2650.

6. Shevtsov, M.B., Streeter, S.D., Thresh, S.J., Swiderska, A., McGeehan, J.E. and Kneale, G.G. (2015) Structural analysis of DNA binding by C.Csp231I, a member of a novel class of R-M controller proteins regulating gene expression. *Acta Crystallogr D Biol Crystallogr*, **71**, 398-407.

7. McGeehan, J.E., Streeter, S.D., Thresh, S.J., Taylor, J.E., Shevtsov, M.B. and Kneale, G.G. (2011) Structural analysis of a novel class of R-M controller proteins: C.Csp231I from *Citrobacter* sp. RFL231. *J Mol Biol*, **409**, 177-188.
